# Construction of a necroptosis-related lncRNA signature for predicting prognosis and revealing the immune microenvironment in bladder cancer

**DOI:** 10.18632/aging.205512

**Published:** 2024-02-05

**Authors:** Jingsong Wang, Zhengyu Jiang, Kai Wang, Qingyuan Zheng, Jun Jian, Xiuheng Liu, Zhiyuan Chen, Rui Yang, Lei Wang

**Affiliations:** 1 Department of Urology, Renmin Hospital of Wuhan University, Wuhan, Hubei 430060, China; 2 Institute of Urologic Disease, Renmin Hospital of Wuhan University, Wuhan, Hubei 430060, China; 3 Department of Urology, People’s Hospital of Hanchuan City, Xiaogan, Hubei 432300, China

**Keywords:** bladder cancer, necroptosis, lncRNA signature, survival, immune microenvironment, chemotherapeutic response

## Abstract

Background: Bladder cancer (BCa) is a common malignancy in the urinary system. Necroptosis, a recently discovered form of programmed cell death, is closely associated with the development and progression of various types of tumors. Targeting necroptosis through anti-cancer strategies has shown potential as a therapy for cancer. We aimed to develop a necroptosis-related lncRNAs (NRlncRNAs) risk model that can predict the survival and tumor immunity of BCa patients.

Methods: We analyzed sequencing data obtained from the TCGA database, and applied least absolute shrinkage and selection operator (LASSO) and Cox regression analysis to identify crucial NRlncRNAs for building a risk model. Using the risk score, we categorized patients into high- and low-risk groups, and assessed the accuracy with the area under the receiver operating characteristic (AUROC) and Kaplan-Meier curves. We performed the RT-qPCR to detect the expression differences of the genes based on the risk model.

Results: We identified a total of 296 NRlncRNAs, and 6 of them were included in the prognostic model. The AUC values for 1-, 3-, and 5-year predictions were 0.675, 0.726 and 0.734, respectively. Our risk model demonstrated excellent predictive performance and served as an independent predictor with high predictive power. Additionally, we performed PCA, TMB, GSEA analyses, and evaluated immune cell infiltration, to reveal significant differences between the high- and low-risk groups in functional signaling pathways, immunological status, and mutation profiles. Finally, we assessed the chemotherapeutic response of several drugs. According to the RT-qPCR results, we found that four NRlncRNAs of the risk model were more highly expressed in BCa cell lines than human immortalized uroepithelial cell line and regulated the occurrence and progression of bladder cancer.

Conclusion: We constructed a novel NRlncRNAs-associated risk model, which could predict the prognosis and immune response of BCa patients.

## INTRODUCTION

Bladder cancer (BCa) is a common malignant tumor of the urinary system. Smoking and occupational exposure to chemical carcinogens are the main risk factors associated with bladder cancer, while other risk factors are less consistently reported [1, 2]. The most predominant pathological subtype is urothelial BCa, which accounts for over 90% of newly diagnosed cases [3]. Around 80% of patients are diagnosed with non-muscle invasive BCa (NMIBC) [4]. Although transurethral resection of bladder tumor (TURBT) has shown great therapeutic effects, NMIBC has a high rate of recurrence and progression after surgical resection [5]. To address this issue, intravesical anti-tumor agents (chemotherapy or immunotherapy) may be given as adjunct treatment after transurethral resection to prevent tumor recurrence and progression in NMIBC [6]. Although immunotherapy has shown promising effects in recent years, it is still at an early stage and has adverse reaction. Therefore, there is an urgent need to identify and establish prognostic markers and therapeutic targets for BCa.

LncRNA, defined as long noncoding RNA, has more than 200 nucleotides that undergoes similar processing as mRNAs [7]. However, lncRNAs lack significant open reading frames. There is growing evidence that lncRNAs take an important part in the gene life cycle as well as cell growth, proliferation, and differentiation [8]. Previous research evidences have shown an intimate association between lncRNAs and the tumorigenicity and metastasis of BCa. For instance, LncRNA RP11-89 has been proven to suppress ferroptosis thereby alleviating the occurrence and progression of BCa, which was expected to serve as a potential therapeutic target for BCa [9]. Another study has reported that LncRNA ELNAT1 may be a hopeful therapeutic target for lymph node (LN) metastatic in BCa, as it could promote lymphangiogenesis and LN metastasis in BCa in a SUMOylation-dependent manner [10].

Necroptosis, a new type of programmed cell death, is regulated by RIPK1-RIPK3-MLKL [11, 12]. This process is involved in pathogen detection and tissue repair. Recent studies suggest that necroptosis participates actively in tumor development, tumor necrosis, tumor metastasis and the tumoral immune response [13]. In breast cancer, RIPK1 could facilitate tumor cell necroptosis to promote metastasis [14]. In colorectal cancer, TRAF6 has been confirmed to stimulate colorectal cell progression through inhibiting the RIPK1/RIPK3/MLKL necroptosis signaling pathway. Therefore, this pathway might represent a new therapeutic target for colorectal cancer [15]. However, the regulatory effects of necroptosis-associated on tumors and its specific mechanisms remain unclear. Thus, identification and exploration of necroptosis-associated lncRNAs (NRlncRNAs) and the identification of prognostic biomarkers among these lncRNAs have enormous potential for clinical application.

In our study, we identified NRlncRNAs with prognostic value to construct a risk model and investigated the correlations between the risk model and the immune microenvironment. We demonstrated that NRlncRNAs with a differential expression in BCa patients could be used as prognostic markers and possible therapeutic targets.

## MATERIALS AND METHODS

### Data collection and preprocessing

We acquired the RNA sequencing (RNA-seq) data and corresponding clinical data of BCa patients from The Cancer Genome Atlas (TCGA) database (https://tcga-data.nci.nih.gov/tcga/). After removing incomplete data and missed survival information, we obtained sequencing and survival data for 406 BCa patients.

### Identification of necroptosis-related lncRNA

We conducted Pearson correlation and co-expression analyses to discern NRlncRNAs with a correlation coefficient greater than 0.4 and a *p*-value less than 0.001. Subsequently, we utilized the “Limma” R package to perform differential expression analysis for the NRlncRNAs exhibiting |logFC| greater than 1 and a corrected *p*-value less than 0.05.

### Establishment of prognostic risk model based on NRlncRNAs

Initially, we employed univariate Cox (uni-Cox) regression analysis to discern lncRNAs that were prognostic with a *p*-value ≤ 0.05. Next, we performed least absolute shrinkage and selection operator (LASSO) regression analysis using a 10-fold cross-validation and a significance threshold of *p* ≤ 0.05 to pick lncRNAs associated with prognosis. Furthermore, the prognostic-associated lncRNAs selected by the LASSO regression analysis were utilized to establish a risk signature based on multivariate Cox (multi-Cox) proportional hazards regression. The calculation formula for the risk score was as follows:


$$\text{Risk}\;\text{score}\;\text{=}\;\sum_{i=1}^{n}\text{coefficient}\;\text{of}\;{\text{NRlncRNA}}_{i}\;\times \;\text{expression}\;\text{of}\;{\text{NRlncRNA}}_{i}$$

On the basis of each patient’s median risk score, we categorized all BCa patients into high- and low-risk groups.

### Assessment and validation of prognostic risk model

Kaplan-Meier analysis was used to compared the survival situation of two risk groups, followed by the long-rank test. We then performed uni- and multi-Cox regression analyses to ascertain the independence of prognostic factors, including the risk score, age, gender, and pathological grade. Finally, we assessed the accuracy of the model based on the area under the receiver operating characteristic (AUROC) and the concordance index (C-index).

### Evaluation of prognostic signature of tumor immune microenvironment

We utilized Gene Set Enrichment Analysis (GSEA) to identify predominantly enriched signaling pathways in the two risk groups. Statistical significance criteria were set at *p* < 0.05 and FDR < 0.25. We subsequently assessed the variations in immune and stromal cell expressions among patients in the two risk groups and calculated StromalScore, ImmuneScore, and ESTIMATEScore (StromalScore + ImmuneScore) for all patients by the “limma” and “estimate” R packages. To evaluate the infiltration of immunocytes and determine the relative content, we employed Single-Sample GSEA (ssGSEA) and the “GSVA” R package. Lastly, we detected the expression levels of immune checkpoints in high- and low-risk groups and the tumor mutation burden (TMB) scores in two risk groups were ascertained according to somatic mutation data.

### Cluster analysis based on prognostic NRlncRNAs

On the basis of NRlncRNA expression levels associated with the risk model, we utilized the “ConsensusClusterPlus” R package to identify potential molecular subgroups responsive to immunotherapy. This analysis ultimately revealed three distinct subforms. Next, we used various packages such as “survival,” “Rzsne,” “limma,” and “reshape2” to estimate and analyze these three distinct subforms.

### Assessing chemotherapy and targeted agent response

To gauge the efficacy of the risk signature in predicting responses to chemotherapy and targeted therapy, we utilized the “oncoPredict” package to examine the correlation between the risk score and the half-maximal inhibitory concentration (IC50) of chemotherapy drugs.

### Cell lines and cell culture

The two BCa cell lines (T24,5637) and the human immortalized uroepithelial cell line (SV-HUC-1) were all bought from American Type Culture Collection (ATCC) (USA). T24 and 5637 were maintained in RPMI 1640 (Hyclone, China) supplemented with 10% fetal bovine serum media. SV-HUC-1 was cultured in Ham’s F-12K (Hyclone, China) with 10% fetal bovine serum media. An incubator with 5% CO2 was applied to culture all cells at 37°C.

### Quantitative real time PCR

We extracted total RNA of two bladder cancer cell lines and human immortalized uroepithelial cell line with TRIzol reagent (Thermo Fisher Scientific, USA). Then we used the reverse transcription kit from Servicebio (China) to obtain cDNAs. After that, the RT-qPCR was conducted on the Quantagene q225 real-time PCR system.

### Statistics analysis

In this study, R software (version 4.2.2) was employed for statistical analyses, and the Wilcoxon rank-sum test was used to assess differences between the two subgroups. Statistical significance was defined as *p* < 0.05 for all two-tailed tests.

### Data availability statement

The datasets of TCGA cohort for this study can be found in the (The Cancer Genome Atlas Program) (https://portal.gdc.cancer.gov/, accessed on 10 July 2023).

## RESULTS

### Identification of NRlncRNAs in BCa patients

**Figure 1:**
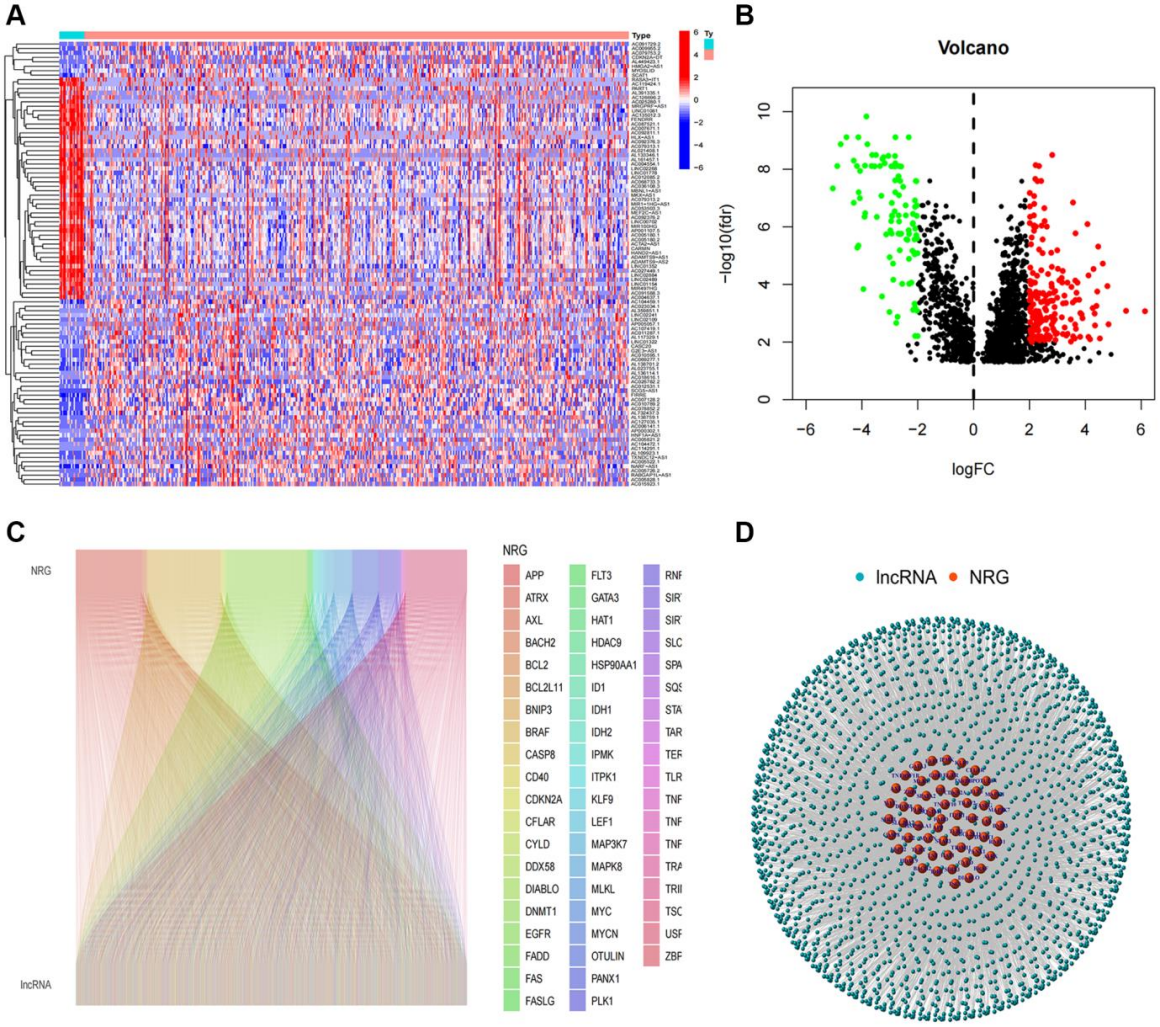
**Identification of necroptosis-related lncRNAs.** (**A**) Heatmap of differentially expressed NRlncRNAs based on their expression levels; (**B**) The volcano diagram of differentially expressed NRlncRNAs; (**C**) Sankey diagram of necroptosis-related genes and lncRNAs; (**D**) The network of necroptosis genes and lncRNAs.

Initially, expression data were retrieved from the TCGA-BLCA database, encompassing 19 normal and 406 tumor samples. A total of 1973 necroptosis-related lncRNAs (NRlncRNAs) were identified, meeting the criteria of a correlation coefficient greater than 0.4 and *p* < 0.001, based on the gene expression matrix. Through a differential expression analysis, we selected 296 NRlncRNAs with |log2 fold change (FC)| > 1 and *p* < 0.05 (Figure 1A, 1B). The correlation between necroptosis-related genes and NRlncRNAs was visually represented in Figure 1C, 1D.

### Construction of the NRlncRNAs risk model

**Figure 2:**
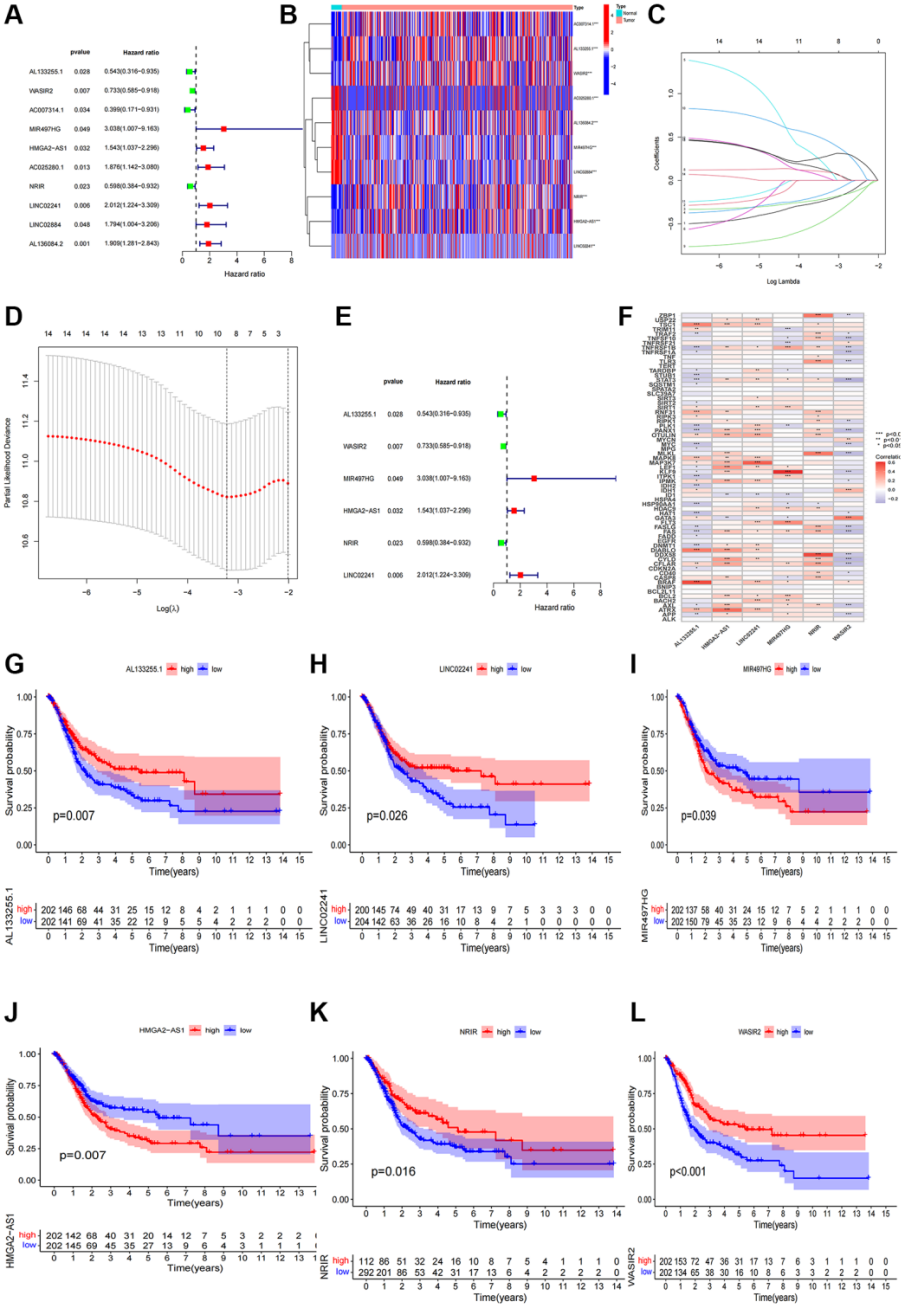
**Construction of the NRlncRNAs risk model.** (**A**) The forest plot of prognostic lncRNAs obtained by uni-Cox regression analysis; (**B**) Heatmap of 15 prognostic lncRNAs expression; (**C**, **D**) The final NRlncRNAs of the risk model was confirmed from LASSO regression analysis; (**E**) The forest plot of six NRlncRNAs of the risk model; (**F**) Correlations between NRlncRNAs of the risk model and necroptosis-related genes; (**G**–**L**) Kaplan–Meier curve of OS analyzed for the six NRlncRNA.

Following a uni-Cox regression analysis, 10 NRlncRNAs related with overall survival (OS) were pinpointed (Figure 2A). Subsequent LASSO and multi-Cox regression analyses led to the identification of 6 NRlncRNAs for the development of a prognostic model (Figure 2B–2E). The correlation between these 6 lncRNAs and necroptosis genes was displayed in Figure 2F. After using the expression levels and regression coefficients of the 6 lncRNAs, each BCa patient’s risk score was computed using the following formula:


Risk score=Al133255.1×( −0.404473)+WASIR×     ( −0.334245)+MIR497HG×(1.298343)+     NRIR×( −0.772492)+LINC02241×     (0.695223)+HIMGA-AS1×(0.380953)

Furthermore, we conducted a survival analysis of the 6 NRlncRNAs and observed their potential as independent prognostic factors for BCa patients (Figure 2G–2L).

### Assessment and validation of the prognostic risk model

**Figure 3:**
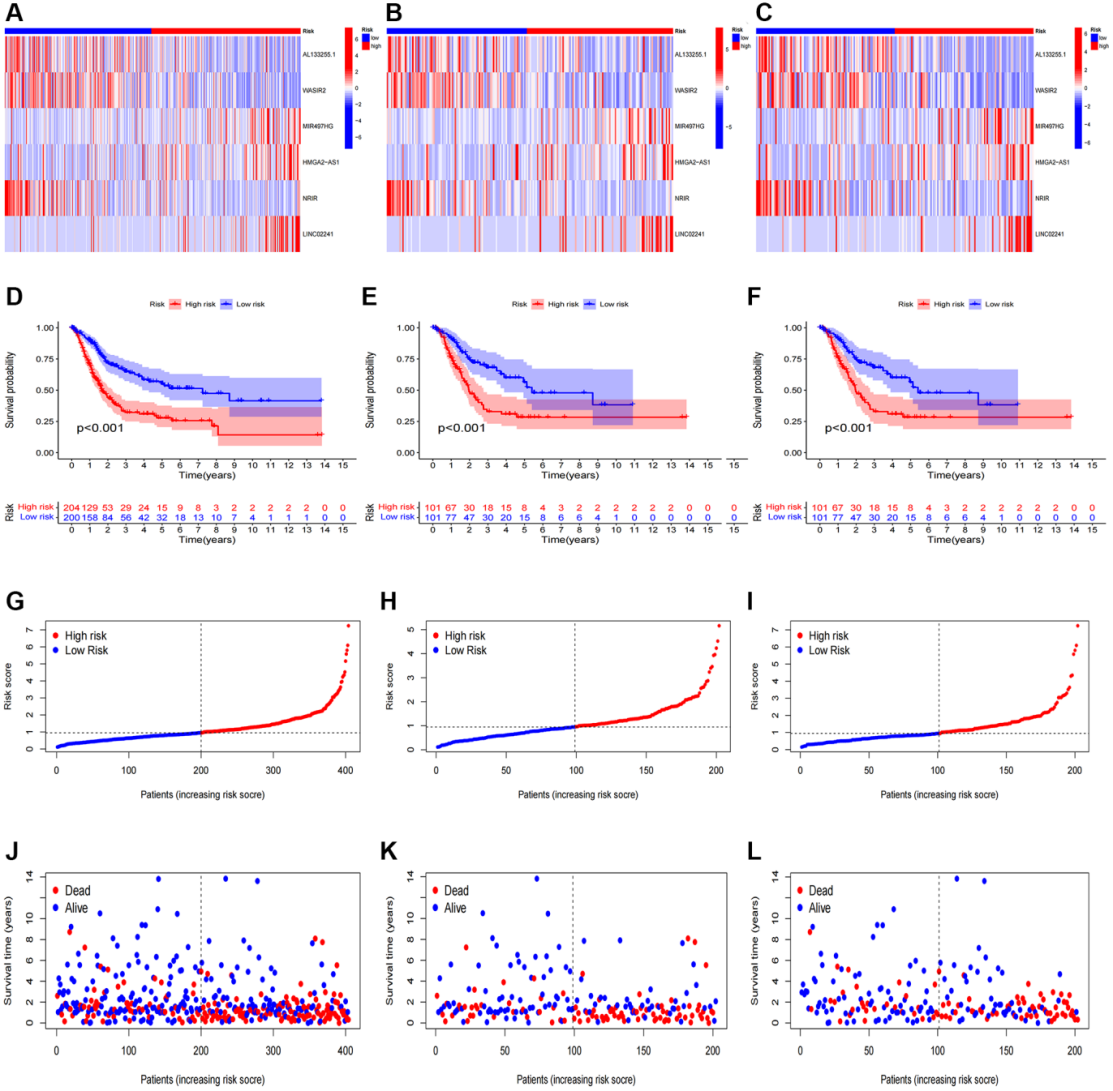
**Prognosis evaluation and internal verification of the risk model.** Heatmaps of six lncRNA expressions (**A**–**C**), risk model (**D**–**F**), survival time and survival status (**G**–**I**), and Kaplan-Meier survival curves of patients (**J**–**L**) in the entire, train, and test cohorts, respectively.

**Figure 4:**
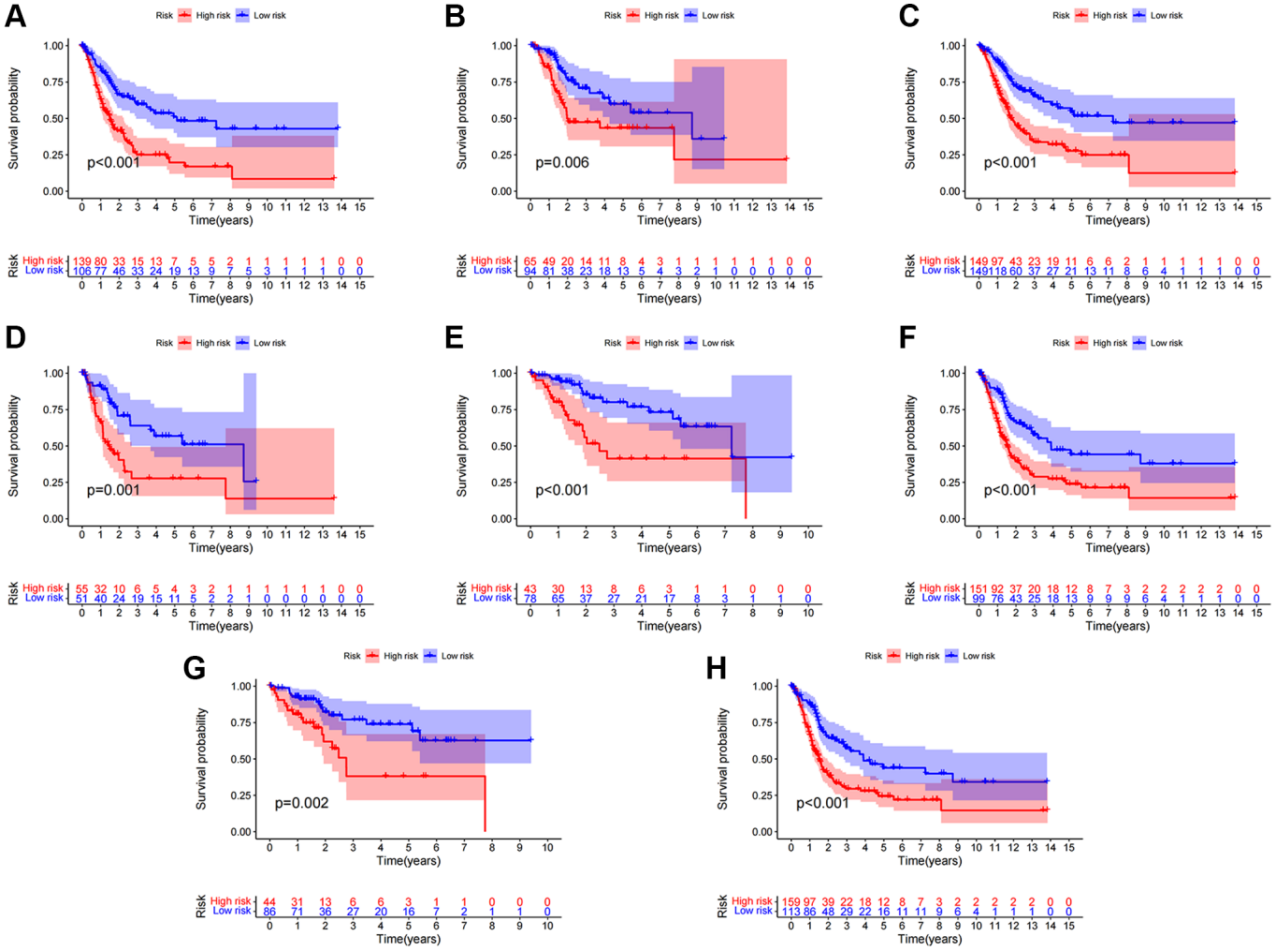
**Clinical significance of the risk model.** (**A**, **B**) Patients with age >65 and age ≤65; (**C**, **D**) Female patients and male patients; (**E**, **F**) Patients with stages I–II and stages III–IV; (**G**, **H**) Patients with stages T1-2 and stages T3-4.

**Figure 5:**
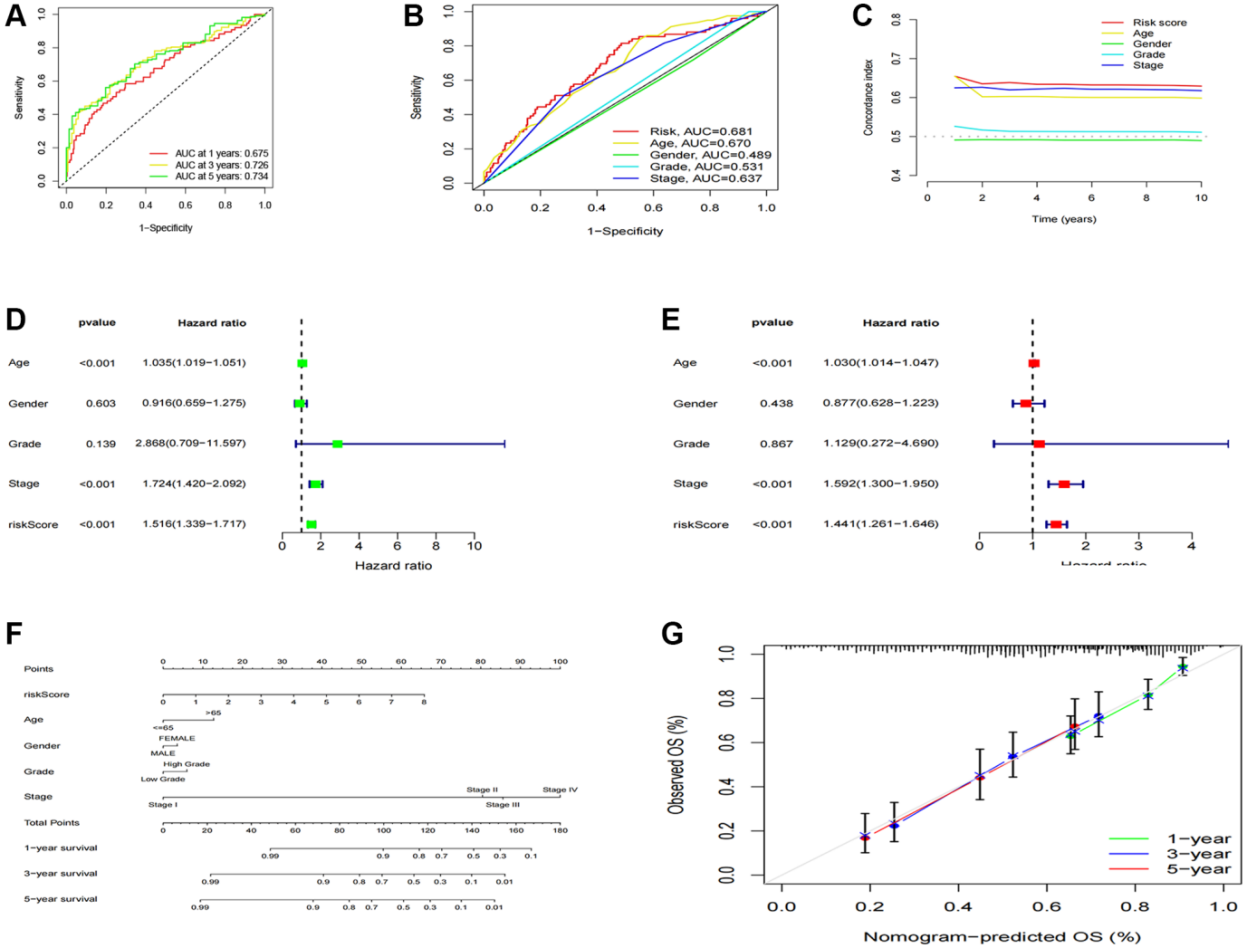
**Construction and validation of the nomogram.** (**A**) 1-, 3-, and 5-years ROC curves of the entire cohort; (**B**) The ROC curves of risk score and clinical features; (**C**) The C-index curves of the risk model; (**D**) Uni-Cox analyses of clinical factors and risk score with OS; (**E**) Multi-Cox analyses of clinical and risk score with OS; (**F**) 1-, 3-, and 5-year OS of BC patients predicted by the nomogram; (**G**) The calibration plots for predicting 1-, 3-, and 5-year OS.

After categorizing all BCa patients into low- and high-risk groups based on their median risk scores, we compared their survival status and time. The results revealed a more favorable prognosis for patients in the low-risk group compared to those in the high-risk group. To confirm the applicability of the risk model, we randomly divided the entire cohort (*n* = 404) into training and testing sets. As expected, consistent with the results in the entire cohort, patients in the high-risk group exhibited worse prognoses compared to those in the low-risk group in both the training and testing sets (Figure 3A–3L). Furthermore, several clinical features, including age (Figure 4A, 4B), gender (Figure 4C, 4D), grade (Figure 4E, 4F), and stage (Figure 4G–4H) confirmed these results. The AUROC values of 1-, 3-, and 5-year survival rates were displayed in Figure 5A. Notably, the AUC value of the risk model surpassed that of individual clinical features (Figure 5B, 5C). Uni- and multi-Cox regression analyses revealed the hazard ratios (HR) of 1.516 and 1.441 for the risk score (Figure 5D, 5E). These findings underscored the significance of the risk score as a robust predictor of prognosis, independent of clinical factors. Moreover, we created a nomogram that integrated the comprehensive landscape of the risk score and independent clinical features to predict 1-, 3-, and 5-year OS (Figure 5F). Calibration charts demonstrated high consistency between actual observation results and the nomogram’s predictions for 1-, 3-, and 5-year OS (Figure 5G). Overall, our results affirm the high accuracy of the risk model in predicting OS for BCa patients.

### Correlations of the risk score with immune factors and the tumor immune microenvironment

**Figure 6:**
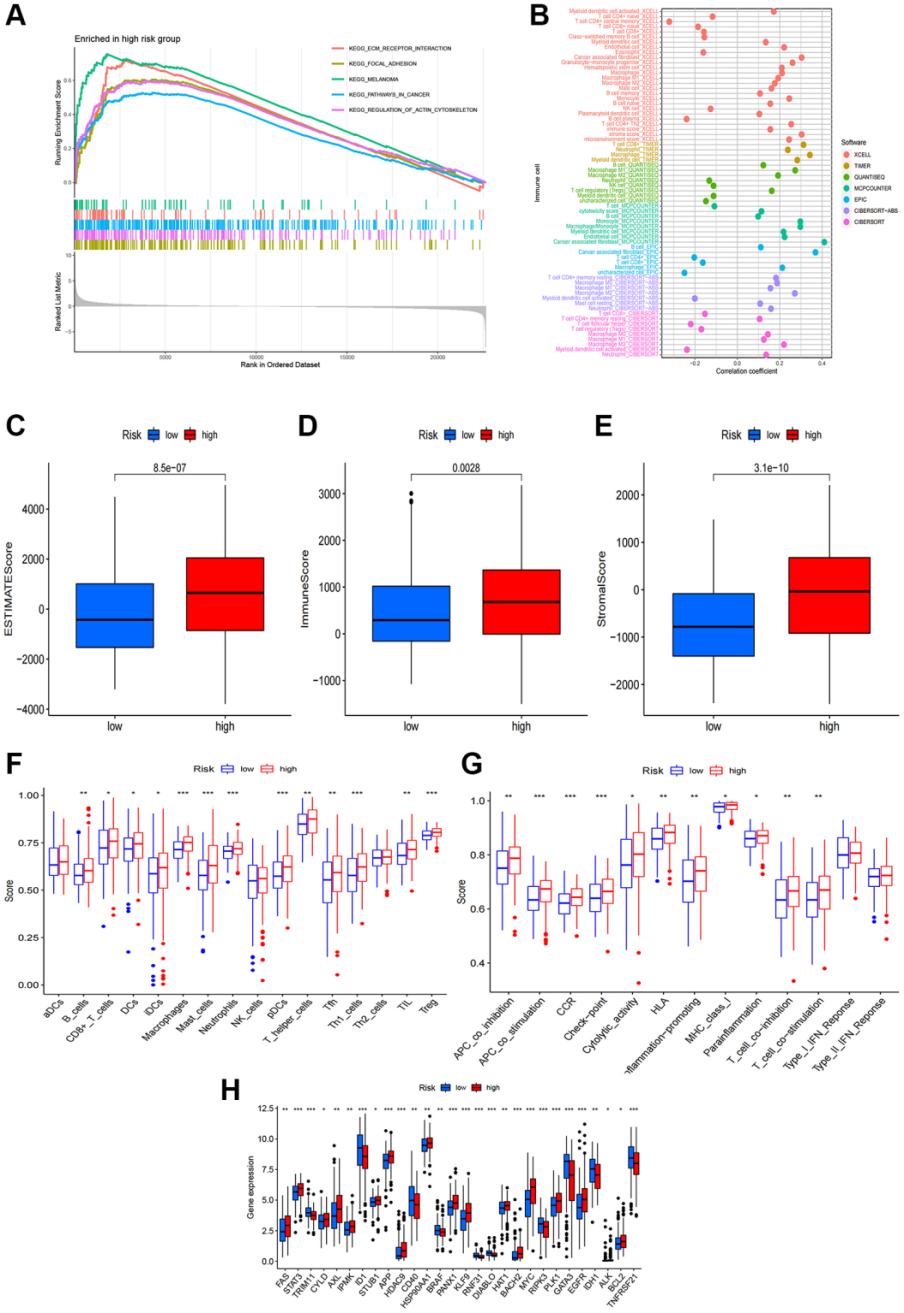
**Correlations of the risk score with tumor immune microenvironment.** (**A**) GSEA of the significantly enriched pathways in the high-risk group; (**B**) The immune cell bubble of risk groups; (**C**–**E**) The boxplots of the comparison of ESTIMATEScore, ImmuneScore and StromalScore, respectively, between low-risk and high-risk groups; (**F**, **G**) The infiltration level of 16 immune cells and 13 immune functions. (**H**) The expression of common immune checkpoints in the risk groups. ^*^p < 0.05, ^**^p < 0.01, ^***^p < 0.001.

We conducted GSEA to explore gene enrichment between two risk groups (Figure 6A). In the high-risk group, we found significant enrichment of ECM receptor interaction, focal adhesion, melanoma, pathways in cancer and regulation of actin cytoskeleton, suggesting a close association with tumor and immune-related pathways. Further investigation into the relationship between risk scores and tumor-infiltrating immunocytes revealed a higher abundance of immunocytes in the high-risk group (Figure 6B). In addition, StromalScore, ImmuneScore, and ESTIMATEScore were all higher in the high-risk group compared to the low-risk group (Figure 6C–6E). To delve deeper into the association between risk scores and immunocytes and functions, we quantified the infiltrating levels of 16 immune cells and 13 immune functions. Our findings demonstrated that most immunocytes exhibited higher scores in the high-risk group, including B cells, CD8+ T cells, DCs, immature dendritic cells, macrophages, mast cells, neutrophils, pDCs, T helper cells, T follicular helper cells, Th1 cells, tumor-infiltrating lymphocyte, and T regulatory cells (Figure 6F). Furthermore, various immune pathways, including APC co-inhibition, APC co-stimulation, CCR, checkpoint, cytolytic activity, human leukocyte antigen, inflammation-promoting, para inflammation, T cell co-inhibition and T cell co-stimulation, scored higher in the high-risk group compared to the low-risk group (Figure 6G). Above conclusions suggested heightened activity of these immune functions in the high-risk group, implying that patients in this group might be more responsive to immunotherapy. We also analyzed the relationship between risk score and immune checkpoints, revealing that certain immune checkpoints, including FAS, STAT3, AXL, KLF9, EGFR, MYC, STUB1, APP were more enriched in the high-risk group while TRIM11, ID1, CD40, RIPK3, GATA3, TNFRSF21 revealed a higher enrichment in the low-risk group (Figure 6H). The elevated expression of these immune checkpoints suggested a potentially enhanced response to immunotherapy for these patients.

### Cluster analysis based on risk signature

**Figure 7:**
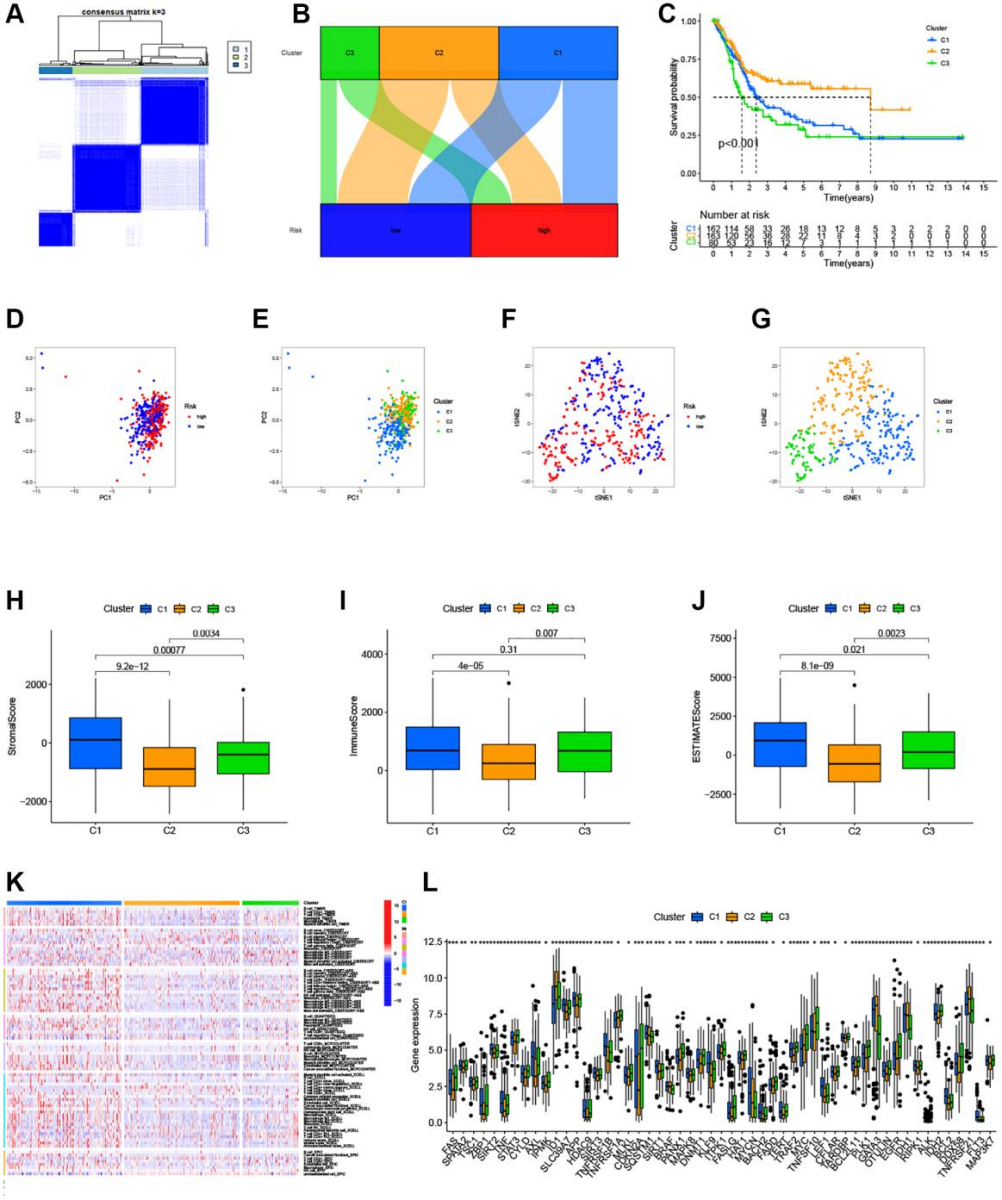
**Clusters analysis for the risk model.** (**A**) BC patients were divided into three clusters according to the consensus clustering matrix (k = 3); (**B**) Sankey diagram of the three clusters; (**C**) Kaplan–Meier curve of OS analyzed for the three clusters; (**D**, **E**) The PCA of risk groups and clusters; (**F**, **G**) The t-SNE of risk groups and clusters; (**H**–**J**) Immune-related scores in clusters; (**K**) Heatmap of immune cells in clusters; (**L**) The expression of immune checkpoints in clusters.

To assess the immune microenvironment and response of different tumor subtypes, we employed cluster analysis to delineate distinct subtypes. Using the “ConsensusClusterPlus” package (Figure 7A), we categorized the patients into three clusters. Our analysis revealed that most patients in the high-risk group were clustered in cluster 1 and 3, while the majority of patients in cluster 2 belonged to the low-risk group (Figure 7B). Furthermore, utilizing survival analysis, cluster 2 exhibited better overall survival compared to clusters 1 and 3 (Figure 7C). To confirm the distinction between these three clusters, we performed PCA and t-SNE, which yielded clear separation of the clusters (Figure 7D–7G). We then conducted immunological factor and tumor microenvironment analyses, which revealed that cluster 1 and 3 had higher Immunescore, Stromalscore, and ESTIMATEscore than clusters 2 (Figure 7H–7J), consistent with our previous results. In addition, we examined the variations in infiltrating immunocytes across the clusters using severe immune platforms and represented them in a heatmap (Figure 7K). Interestingly, we found that a majority of immune checkpoints had lower expression in cluster 2 (Figure 7L).

### Tumor mutation burden (TMB) analysis

**Figure 8:**
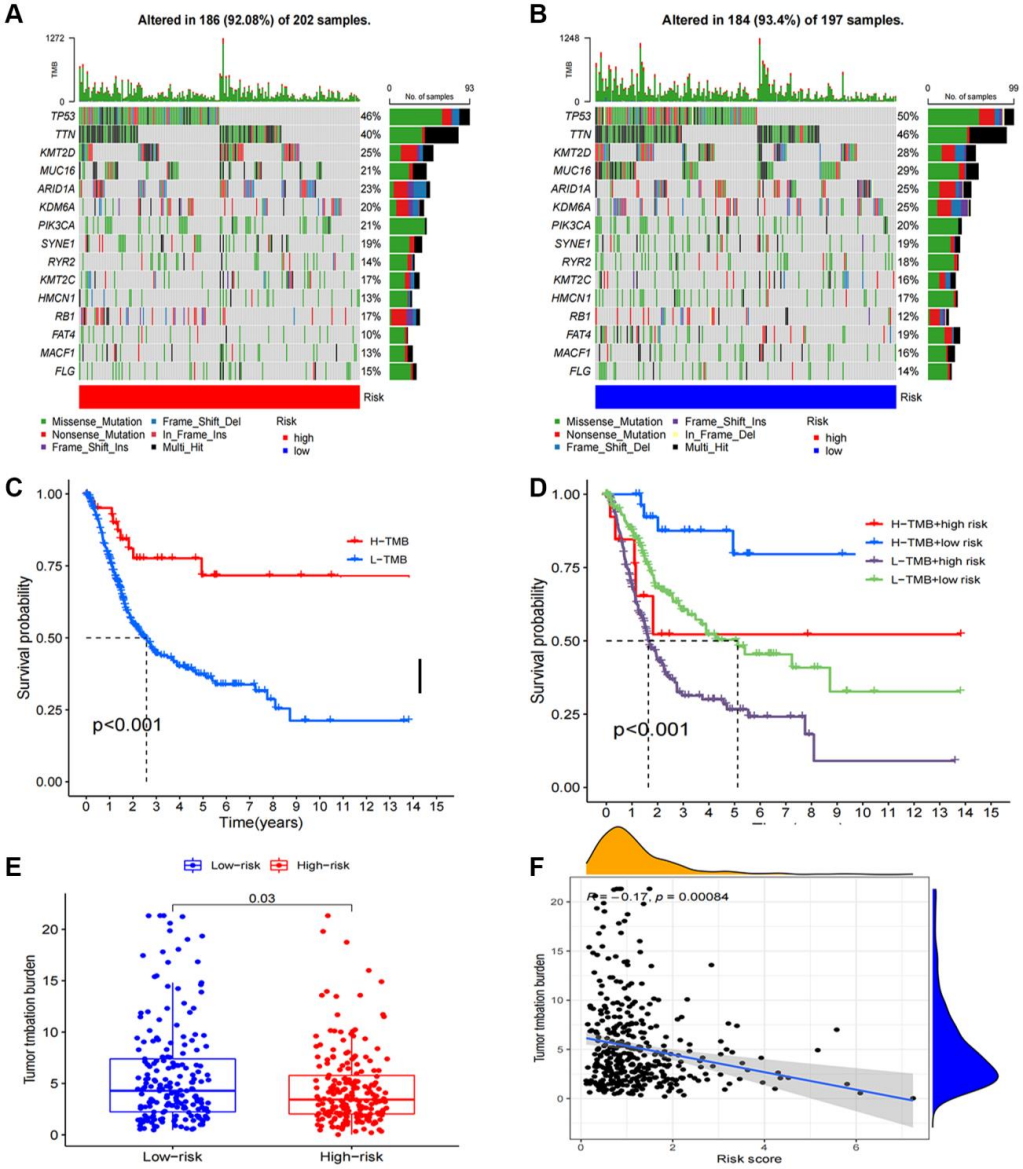
**Tumor mutation burden calculation analysis.** (**A**, **B**) Tumor somatic mutation waterfall graph in high-risk group and low-risk group; (**C**) Kaplan–Meier curve of OS analyzed for TMB; (**D**) Kaplan–Meier curve of OS analyzed for TMB and risk groups; (**E**) The relationship between TMB and risk groups; (**F**) Correlation analysis of the risk score and TMB.

In our study, we found that 184 (93.4%) of 197 samples in the low-risk group exhibited a wider TMB compared to the high-risk group (92.08%), suggesting that immunotherapy might have a greater impact on the low-risk group. Furthermore, prominent genetic changes were noted in genes such as PIK3CA, TP53, TTN, and MUC16, each with a mutation frequency exceeding 15% (Figure 8A, 8B). Based on the survival curves, the low-TMB group showed superior overall survival than the high-TMB group (Figure 8C). Additionally, a robust correlation between TMB and risk score was evident, as demonstrated in Figure 8E, 8F (R = 0.13, *p* = 0.016). Combining these two parameters, we observed that patients who exhibited the lowest risk score and lowest TMB resulted the best prognosis, while those with the highest risk score and highest TMB resulted the worst prognosis (Figure 8D).

### Potential effects of chemotherapy in BCa

**Figure 9:**
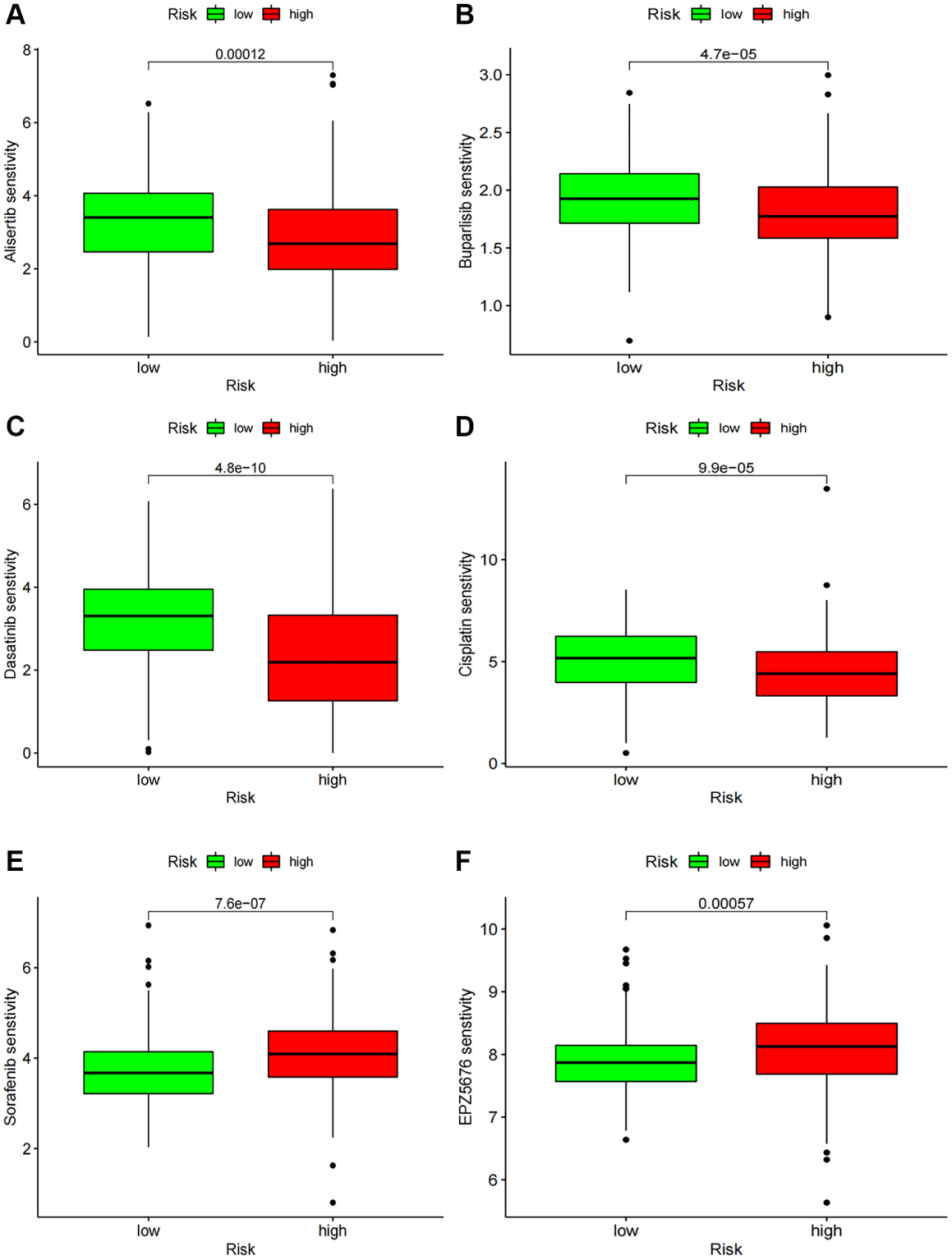
**Chemotherapy drug sensitivity.** (**A**–**F**) IC50 of several chemotherapy drugs in BC. (**A**) Alisertib; (**B**) Buparlisib; (**C**) Dasatinib; (**D**) Cisplatin; (**E**) Sorafenib; (**F**) EPZ5676.

We assessed the sensitivity of chemotherapy drugs in BCa patients by determining their IC50 values. Our findings revealed that the IC50 values for Alisertib, Buparlisib, Dasatinib, and Cisplatin were lower in the high-risk group, implying a correlation between the high-risk status and increased sensitivity to these drugs. Conversely, patients with low-risk status exhibited significantly lower IC50 values for Sorafenib and EPZ5676 (Figure 9A–9F).

### Expression of prognostic NRlncRNAs in bladder cancer

**Figure 10:**
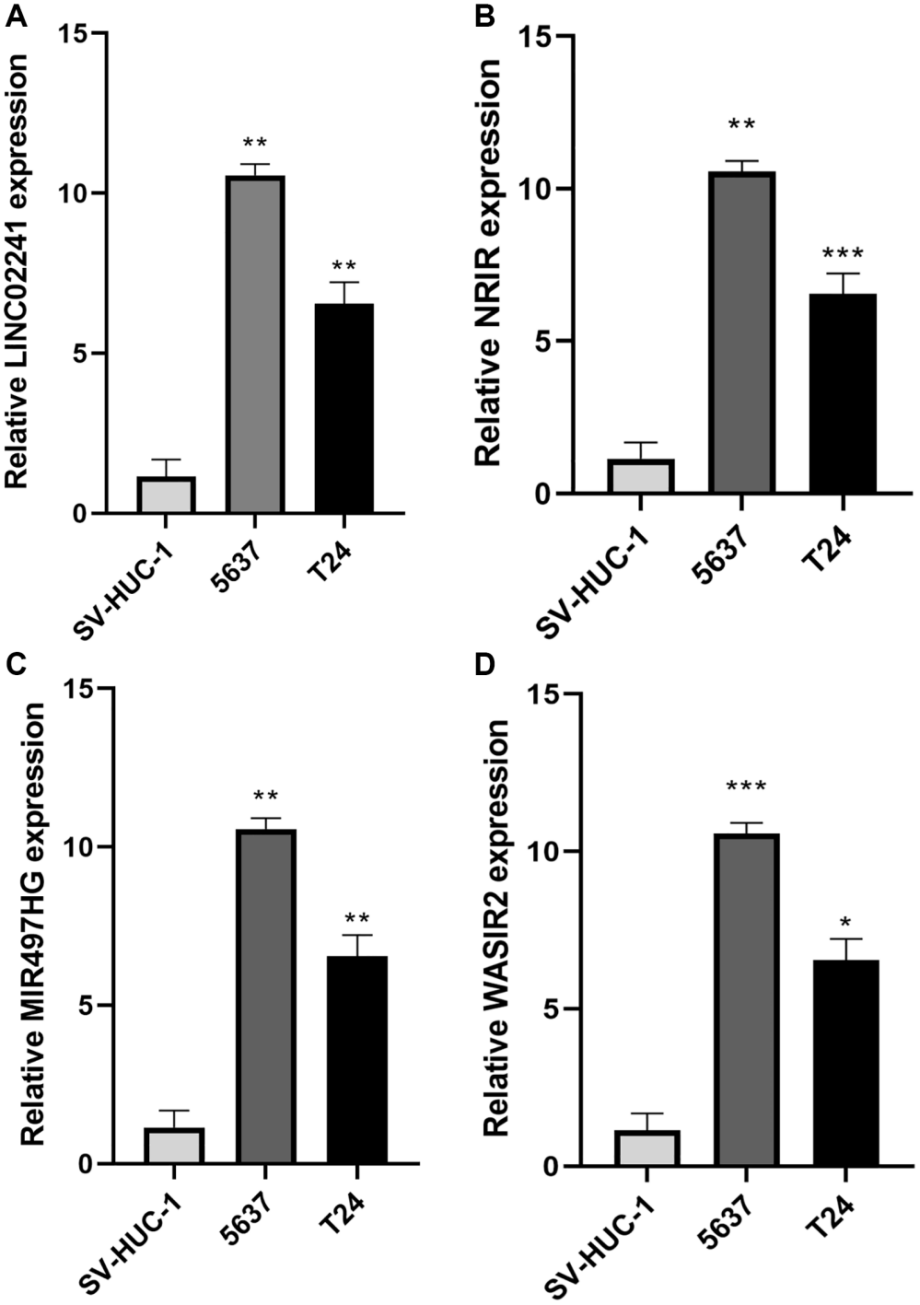
**Expression of necroptosis-related genes in bladder cancer.** (**A**–**D**) Relative expression of LINC02241, NRIR, MIR497HG and WASIR2 in 2 BCa cell lines (5637, T24) and SV-HUC-1 cell line. ^*^p < 0.05; ^**^p < 0.01; ^***^p < 0.001.

We conducted RT-qPCR to detect the expression levels of NRlncRNAs in the risk model. The outcomes revealed that LINC02241, NRIR, MIR497HG and WASIR2 exhibited higher expression in BCa cell lines compared to normal urinary epithelial cells (Figure 10A–10D).

## DISCUSSION

As widely recognized, resistance of cell apoptosis is a major cause of chemotherapy failure during cancer treatment [16]. Necroptosis, an alternative form of regulated necrotic cell death independent of caspases, and acts as an alternative mode for overcoming resistance to programmed cell death via apoptosis [17]. Necroptosis has a dual role in tumors. On the one hand, key mediators in the necroptosis pathway involve the promotion of tumor progression and metastasis [18, 19], while on the other hand, necroptosis can protect against tumor development when apoptosis is compromised [20].

While numerous studies have focused on developing lncRNA signatures for assessing cancer prognosis [21, 22], limited research has explored the role of NRlncRNAs in the tumor microenvironment and prognosis. In our study, we explored the intricate correlation between the tumor microenvironment, immune cell infiltration, immunological checkpoints, and NRlncRNAs. Our findings might provide valuable insights for the clinical diagnosis and treatment of BCa.

First, we collected 296 NRlncRNAs with varying levels of expression. Using LASSO, uni- and multi-Cox regression analyses, we identified 6 NRlncRNAs (ALI33255.1, WASIR2, MIR497HG, HMGA2-AS1, NRIR, and LINC02241) and built a risk model for NRlncRNAs. The LASSO algorithm calculated the risk score for the OS outcome of each patient, to classify all BCa patients into high- and low-risk groups. Notably, patients in the high-risk group exhibited a worse prognosis than their low-risk counterparts. The risk model demonstrated favorable discrimination performance in predicting BCa patient prognosis. Furthermore, our findings were aligned with prior studies emphasizing the importance of lncRNA-related signatures in BCa prognosis prediction [23–25]. Our model exhibited well sensitivity in predicting BCa patient survival, as evidenced by clinicopathological analysis, survival analysis, PCA, and TMB analysis.

Recent studies have emphasized the integral relationship between tumor immune microenvironment and tumor initiation and progression [26–28]. Immunocyte infiltration serves as a regulator of tumor progression, influencing prognosis by reshaping the tumor microenvironment [29]. In our study, we observed an interrelation between the infiltration levels of certain immunocytes and the risk score. Additionally, using the “ConsensusClusterPlus” package, patients were classified into three clusters based on our risk model. Cluster 2 had significantly reduced immunological checkpoint expression compared to clusters 1 and 3, suggesting an association between consensus clustering and immunological microenvironment. Since immunotherapy has proven to be an important treatment for BCa, identifying patients who can derive maximum benefit from immunotherapy remains crucial [30]. TMB, reflecting the number of somatic mutations per megabase of genomic sequence, aids in identifying cancer patients likely to respond to immune checkpoint inhibitors [31]. Our results showed that patients that possessed the lowest TMB experienced the best prognosis and were more likely to benefit from chemotherapy drugs. In summary, these findings might offer a promising method for optimizing immunotherapy for BCa patients.

Our study still had some limitations. First, we were unable to perform external validation analysis, as the 6 identified lncRNAs could not be found in other databases. Second, while our study primarily relied on bioinformatics analysis, further experiments were necessary to establish the mechanism of the NRlncRNAs in BCa.

In conclusion, our study unveils a novel signature crafted from NRlncRNAs, predicting survival in BCa patients, and offering insights into the immune microenvironment and chemotherapy treatment.
